# Inflammation-Induced Mucosal KYNU Expression Identifies Human Ileal Crohn’s Disease

**DOI:** 10.3390/jcm9051360

**Published:** 2020-05-06

**Authors:** Meik Huhn, Martina Herrero San Juan, Balint Melcher, Caroline Dreis, Katrin G. Schmidt, Anja Schwiebs, Janet Collins, Josef M. Pfeilschifter, Michael Vieth, Jürgen Stein, Heinfried H. Radeke

**Affiliations:** 1 Goethe Universität, pharmazentrum frankfurt/ZAFES, Institute of Pharmacology and Toxicology, Hospital of the Goethe University, 60590 Frankfurt/Main, Germany; huhn@em.uni-frankfurt.de (M.H.); herrero@em.uni-frankfurt.de (M.H.S.J.); dreis@em.uni-frankfurt.de (C.D.); ks.schmidt86@gmail.com (K.G.S.); schwiebs@med.uni-frankfurt.de (A.S.); pfeilschifter@em.uni-frankfurt.de (J.M.P.); 2 Institute of Pathology, Klinikum Bayreuth, 95445 Bayreuth, Germany; melcherb@gmail.com (B.M.); vieth.lkpathol@uni-bayreuth.de (M.V.); 3 Interdisciplinary Crohn-Colitis Center Rhein-Main, Schifferstrasse 59, 60594 Frankfurt/Main, Germany; collins@posteo.de (J.C.); j.stein@em.uni-frankfurt.de (J.S.)

**Keywords:** kynurenine, kynureninase, 3-hydroxyanthranilic acid, Crohn’s disease, tryptophan, IDO1

## Abstract

The widely varying therapeutic response of patients with inflammatory bowel disease (IBD) continues to raise questions regarding the unclarified heterogeneity of pathological mechanisms promoting disease progression. While biomarkers for the differentiation of Crohn’s disease (CD) versus ulcerative colitis (UC) have been suggested, specific markers for a CD subclassification in ileal CD versus colonic CD are still rare. Since an altered signature of the tryptophan metabolism is associated with chronic inflammatory disease, we sought to characterize potential biomarkers by focusing on the downstream enzymes and metabolites of kynurenine metabolism. Using immunohistochemical stainings, we analyzed and compared the mucosal tryptophan immune metabolism in bioptic samples from patients with active inflammation due to UC or CD versus healthy controls. Localization-specific quantification of immune cell infiltration, tryptophan-metabolizing enzyme expression and mucosal tryptophan downstream metabolite levels was performed. We found generally increased immune cell infiltrates in the tissue of all patients with IBD. However, in patients with CD, significant differences were found between regulatory T cell and neutrophil granulocyte infiltration in the ileum compared with the colon. Furthermore, we observed decreased kynurenine levels as well as strong kynureninase (KYNU) expression specifically in patients with ileal CD. Correspondingly, significantly elevated levels of the kynurenine metabolite 3-hydroxyanthranilic acid were detected in the ileal CD samples. Highlighting the heterogeneity of the different phenotypes of CD, we identified KYNU as a potential mucosal biomarker allowing the localization-specific differentiation of ileal CD versus colonic CD.

## 1. Introduction

In clinical practice, the therapeutic response of patients with ileal Crohn’s disease (iCD) frequently differs from that of patients with extensive CD-associated inflammation of the colon. In 2019, Dulai et al. hypothesized that Crohn’s disease could be reclassified into ileum-dominant or isolated colonic CD subtypes [1]. For example, clinical outcomes under therapy with the monoclonal tumor necrosis factor alpha (TNFα) antibody, infliximab, are generally superior in patients with colonic CD (cCD) compared with iCD [2,3]. Furthermore, studies based on a variety of serological [4], clinical [5], microbial [6] and genetic [7,8,9] markers have described a corresponding heterogeneity in patterns of involvement in CD. Although disease localization has been unequivocally linked to differing clinical outcomes in patients with CD, the mechanisms behind this differential pathology are still unclear. In this context, the identification of a biomarker capable of clearly differentiating the phenotypes of CD would represent a crucial step on the way to an individualized, patient-specific therapy optimized to achieve complete mucosal healing, a central therapeutic goal of CD therapy.

Nikolaus et al. were able to show a decrease in tryptophan (TRP) and an increase in kynurenine (Kyn) concentrations in the serum of patients with inflammatory bowel disease (IBD). In addition, indoleamine 2,3-dioxygenase (IDO1) mRNA expression has been shown to be elevated in inflamed tissue of patients with CD and ulcerative colitis (UC) [10]. In addition, Gupta et al. observed a correlation between TRP metabolism and the CD activity [11]. Correspondingly, immigration of innate immune cells into inflamed regions of the gastrointestinal system has been described as the main source of TRP-degrading enzymes and producers of TRP downstream metabolites [12,13]. Thus, the TRP metabolization arsenal is carried to the site of inflammation by immune cells. However, it remains unclear (1) whether IDO1 is expressed in an activated state, (2) how high the local mucosal Kyn concentrations are and (3) how further degradation of Kyn into TRP downstream metabolites is regulated.

TRP immune metabolism is strongly involved in keeping the intestinal barrier intact and preventing inflammation by mediating regulatory functions affecting regulatory T cells (Treg) and Th17 adaptive immunity [14,15]. It is thought that this immunoregulatory function is performed by IDO and its first downstream TRP metabolite Kyn. In principle, the majority of publications describe Kyn as a regulator of immunosuppression [16,17]. Kyn has been shown to interact with the intracellular transcription factor aryl hydrocarbon receptor (AHR), thereby suppressing effector T cells by regulating expression of cytotoxic T-lymphocyte-associated protein 4 and programmed death protein 1 as well as promoting the differentiation of Treg [18,19,20]. It remains unclear why, despite increased expression of IDO1 and the systemic elevation of Kyn, highly active inflammatory processes nevertheless persist in the pathology of IBD.

Whereas both TRP and Kyn, as well as the corresponding TRP-metabolizing enzymes IDO1 and tryptophan 2,3-dioxygenase (TDO) have been examined in some detail, downstream enzymes and metabolites of the TRP metabolism have not been closely studied. In this context, Harden et al. revealed an increase in the expression of the Kyn-degrading enzyme kynureninase (KYNU) in the Th1/Th17 lymphocyte-driven skin disease psoriasis as well as in the general clinical manifestations of chronic inflammation-related diseases [21]. The authors concluded that KYNU may act as a dichotomous modulator of immune responses, regulating the switch between immunosuppression and inflammation. While in cancer, IDO1 is preferentially upregulated, in chronic inflammatory diseases such as psoriasis and atopic dermatitis, elevation of KYNU was found to be more pronounced than that of IDO1 [21]. Whether KYNU is increased in IBD, and what function this Kyn-degrading enzyme has in the development and course of chronic inflammatory disorders, is still unclear. This raises the question of whether the inflammation-related pathologies arise due to a decrease in local Kyn concentration, or whether they are caused by an increase in the potentially proinflammatory downstream TRP metabolite 3-hydroxyanthranilic acid (3-HAA) [21,22,23].

The aim of this study was to investigate the local mucosal TRP immune metabolism with a special focus on the enzyme KYNU. Additionally, we performed a detailed comparison of site-specific inflammation-induced immune cell infiltration in patients with IBD. We found differences in the expression pattern of KYNU in the different CD phenotypes and a decreased level of the immunosuppressive TRP metabolite Kyn in iCD. Furthermore, we identified distinct characteristic immunological profiles for iCD and cCD. In summary, we characterized KYNU as a potential new marker and therapeutic target for iCD as regulator of the ratio of pro- and anti-inflammatory TRP downstream metabolites.

## 2. Methods

### 2.1. Ethical Approval

This research was performed in line with the principles of the Declaration of Helsinki. The study (309_19 Bc) was approved by the ethical committee Friedrich-Alexander-Universität Erlangen-Nürnberg.

### 2.2. Patient and Tissue Sample Characteristics

In order to characterize immune cell markers, enzymes and metabolites of the TRP metabolism through immunohistochemistry (IHC), we included three different cohorts of patients with IBD and defined them as control, CD and UC (Table 1). Controls were defined as individuals that were scoped within the framework of colorectal cancer screening but had neither symptoms, nor polyps nor inflammatory lesions. The CD cohort was subclassified according to disease localization by differentiating patients with iCD from those with cCD. In both IBD cohorts, CD and UC, biopsies were collected from patients with an active disease state and obtained from the bowel region showing maximally severe inflammation as judged by the endoscopist. In the CD cohort, 14 samples were collected from 12 patients. Nine of these tissue samples showed small bowel inflammation, whereas five revealed colonic inflammation. In the UC cohort of 11 patients, as expected, all samples showed inflammation of the large intestine. In this study setting, one biopsy was taken per individual (patient/control) and location (small intestine/large intestine). Thus, a maximum of two biopsies (small intestine + large intestine) per patient/control were examined. The inflammatory state of the intestinal tissue samples was validated by a pathologist and determined using the Riley score [24], a four-point scale in which a score of 1 identifies disease remission and 2 or more indicates active disease. An important criterion leading to the choice of the Riley score in preference to other histological indices such as the Nancy score was the possibility of differentiating healthy controls (Riley score = 0) from patients in remission (Riley score = 1), in order to exclude the latter from study participation. Determinant factors of the Riley score are acute or chronic inflammatory cell infiltrates, crypt abscesses, mucin depletion, integrity of surface epithelium and crypt architectural irregularities. The CD cohort showed on average a higher Riley score (3.0) than the UC cohort (2.3). Control biopsies (9 small intestine, 12 large intestine) were collected from 12 healthy individuals. None of the control samples showed any signs of inflammation (Riley score 0; Table 1).

### 2.3. Immunohistochemistry

Immunohistochemical staining was performed with formalin-fixed and paraffin-embedded sections (2 μm). Xylene deparaffinization and graded alcohol hydration steps were followed by citrate-dependent (pH6) protein antigen retrieval (Dako, Santa Clara, CA, USA). After several washes, endogenous peroxidase was blocked using 0.3% hydrogen peroxide and 0.1% sodium azide diluted in PBS. To block signals from endogenous avidin, biotin and biotin-binding proteins, we used an avidin/biotin blocking kit (Vector Laboratories, Burlingame, CA, USA). Nonspecific binding was blocked with protein block (Dako, Santa Clara, CA, USA) or 2% goat serum in antibody diluent (Dako, Santa Clara, CA, USA) at room temperature. Sections were then incubated overnight at 4 °C with the primary antibody diluted in antibody diluent (Dako, Santa Clara, CA, USA). A negative control section was included, in which only the secondary antibody (anti-mouse/anti-rabbit) was used (Figure S2). All primary antibodies and corresponding dilutions are listed in Table S1. After two washing cycles, an anti-mouse or anti-rabbit HRP-envision system (Nichirei Biosciences, Tokyo, Japan) and DAB-chromogen (Vector Laboratories, Burlingame, CA, USA) was applied to visualize the stainings. Tissue sections were counterstained with Meyers hematoxylin (PanReac AppliChem, Darmstadt, Germany) and mounted with Roti-Mount Aqua (CarlRoth, Karlsruhe, Germany). Staining results were documented by Olympus inverted microscopy (Olympus K. K., Tokyo, Japan). Semiquantitative interpretation of positive areas/stained cells was conducted using the BZ Analyzer Software 9000 with its specific IHC-based algorithm (Keyence, Osaka, Japan). Positive staining was defined as clear brown staining. We determined the number of positive cells in each section per biopsy and calculated the percentage of positive area per total area (for CD3, CD68, CD11b, CD11c, MPO, IDO, TDO, KYNU, Kyn and 3-HAA) or positive cells per total area (KMO and FOXP3). A corresponding example is shown in Figure S1. Quantification was performed by a blinded independent examiner who was unaware of the diagnosis and histopathological scoring. All relevant target structures were stained in each biopsy and included in the statistics. All histology specimens were matched with corresponding samples of each of the other cohorts taken from the same bowel segment.

### 2.4. Primary Cell Isolation of Antigen-Presenting Cells and CD3+ T Cells

Monocytes, macrophages, dendritic cells, B cells and CD3+ T cells were isolated from Pperipheral blood mononuclear cells (PBMCs) using Ficoll-Histopaque 1077 gradients (Sigma-Aldrich, Steinheim, Germany). For the isolation of monocytes (Catalog#19058), B cells (Catalog#17954) and CD3+ T cells (Catalog#17911), negative selection was performed using EasySep Human Cell Isolation Kits (Stemcell Technologies, Vancouver, BC, Canada). Macrophages and dendritic cells were differentiated as described in [25,26].

### 2.5. Cultivation and Stimulation of Antigen-Presenting Cells and CD3+ T Cells

Monocytes, macrophages, dendritic cells, B cells or CD3+ T cells were isolated from PBMC as described. Cells were seeded out at a density of 1 × 10^6^/mL and left untreated in RPMI medium (Greiner bio-one, Frickenhausen, Germany), 1% HEPES buffer solution, 100 IU/mL penicillin and 100 IU/mL streptomycin. Cells were cultivated overnight with human AB serum (Sigma-Aldrich, Steinheim, Germany) in a final concentration of 1% (v/v) in RPMI medium. For stimulation, cells were cultivated for 24 h in serum-free RPMI medium, 100 U/mL IFNγ (PeproTech, Hamburg, Germany), 100 ng/mL TNFα (PeproTech, Hamburg, Germany) and 10 ng/mL IL-1β (PeproTech, Hamburg, Germany). All reagents were diluted in PBS/0.1% BSA.

### 2.6. RNA Isolation, cDNA Synthesis and Real-Time Quantitative PCR

Isolated monocytes, macrophages, dendritic cells, B cells and CD3+ T cells were pelleted, and total RNA was isolated using Isolate II RNA Micro Kit (Bioline, Heidelberg, Germany) according to the manufacturer’s instructions. Equal RNA amounts were transcribed into cDNA by reverse transcriptase with the Precision nanoScript Reverse Transcription Kit (Primerdesign, Southampton, UK), and a standard real-time (RT)-PCR program (65 °C, 5 min; 55 °C, 20 min; 75 °C, 15 min) was used. For real-time quantitative PCR (qRT-PCR) mRNA analysis, 5′FAM labeled testing probes of housekeeping genes GAPDH and RPL13A (Primer Design, Southampton, UK), IDO1 (Hs00984148_m1) and KYNU (Hs01114105_m1) were used in the complete reaction mixtures. All probes were purchased from Applied Biosystems (Foster City, CA, USA) in a final concentration of 250 nM. The qRT-PCR analyses were performed in technical duplicates with 5 µL Precision FAST 2x- qPCR Master-Mix (Primer Design, Southampton, UK), 3.5 µL H2O, 0.5 µL 5′FAM marked testing probe and 1 µL cDNA. The following program was used: 95 °C for 5 min, then 3 s at 95 °C and 30 s at 60 °C (40×), alternately. Relative mRNA expression was calculated based on the normalized ratio of nonregulated GAPDH and RPL13A expression and the 2^-ΔCt^ method.

### 2.7. Statistical Analysis

Statistical analysis was performed using GraphPad Prism version 6 (GraphPad Software, La Jolla, CA, USA). For the comparison of healthy controls versus inflamed IBD samples and iCD versus cCD samples, Mann–Whitney or Kruskal–Wallis tests were applied for nonparametric distribution. Nonparametric correlation between the Riley score and/or protein/metabolite expression was tested with Spearman’s rho. For the in vitro experiments, the two-tailed Student’s *t*-test was applied. In all figures, asterisks (*) denote significant comparisons, with * for *p* ≤ 0.05, ** for *p* < 0.01, *** for *p* < 0.001 and **** for *p* < 0.0001. All data are presented as mean ± SD. The outlier calculator from GraphPad was used for all analyses, and significant outliers were removed.

## 3. Results

### 3.1. Local Intestinal Tissue Inflammation in Patients with CD Is Characterized by Immune Cell Infiltrates and Varies According to Anatomic Localization

**Figure 1:**
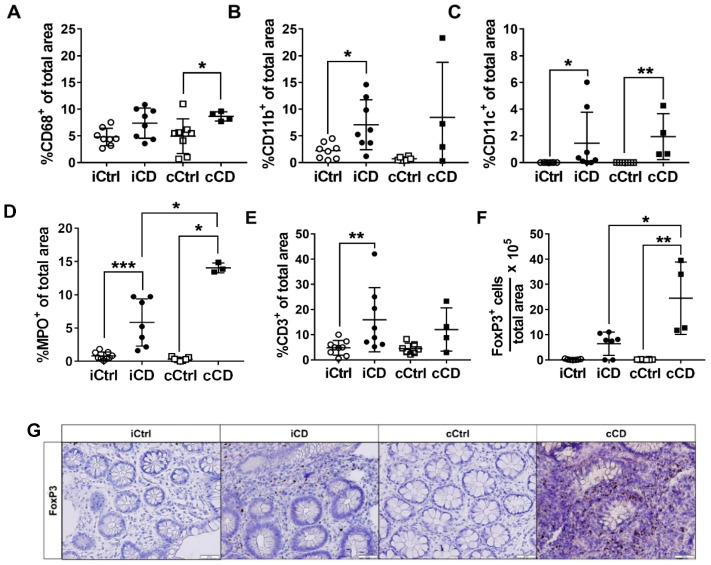
Enhanced immune cell infiltration in intestinal tissue of patients with active CD. Differential quantitative and localization-dependent evaluation of the area percentages of (**A**) CD68+, (**B**) CD11b+, (**C**) CD11c+, (**D**) MPO+, (**E**) CD3+ and (**F**) FoxP3+. (**G**) Analysis of FoxP3 expression in paraffin-embedded tissue samples of patients with CD (representative images, magnification 20×). Inflamed tissue of patients with iCD and cCD were compared with noninflamed control (ctrl) samples. For the analysis of FoxP3, we determined the ratio of counted cells to the total area (µm^2^) and multiplied with 10^5^ for better visualization. (**A**–**F**) Data are shown as mean ± SD of *n* = 4–9 for different patient groups and controls; * for *p* ≤ 0.05, ** for *p* < 0.01, and *** for *p* < 0.001 using the Mann–Whitney test. cCD = colonic CD, cCtrl = colon non-IBD control, iCD = ileal CD, iCtrl = ileum non-IBD control.

Following their classification according to the Riley score, the intestinal biopsies were subjected to immunohistochemical screening for detection of immune cell infiltrates in order to characterize the type of inflammation. The aim of this analysis was to determine the extent to which TRP-relevant enzymes are carried to the site of inflammation by immune system cells and to compare in detail the mucosal immunological profiles of patients with CD inflammation affecting different bowel regions (iCD vs. cCD). As a first step, we performed a general comparison of UC and CD tissue. However, as mentioned above, our main focus was to compare ileal and colonic inflammation, the two major phenotypes of disease manifestation in CD. We determined and compared the number of CD68-, C11b-, CD11c-, myeloperoxidase (MPO)-, CD3- and FoxP3-expressing cells in the tissue of patients with CD versus controls (Figure 1). In the tissue of healthy controls, positive signals of any of these immune cell markers were found only in the subepithelial layer, with the exception of CD11b, which was also detected within the layer of crypt cells. In contrast, crypts in the inflamed tissue of patients with IBD occasionally showed positive signals for intraepithelial CD3 (IEL). In inflamed IBD tissue, CD68, CD11b, CD11c and FoxP3 were detected exclusively in the subepithelial layer (Figure S3A). The number of antigen-presenting cells (APC) was found to be similar for iCD and cCD. However, significant differences in the percentage of CD68+ cells were detected between cCD and colon controls (Figure 1A). Further analyses revealed a significant increase in CD11b+ cells in inflamed iCD tissue compared to healthy controls (Figure 1B). The percentage of CD11c+ cells, another indicator for tissue APC, was found to be significantly elevated in inflamed iCD and cCD tissue compared to controls (Figure 1C). MPO, a marker for neutrophil granulocytes, was elevated in both types of CD (iCD and cCD) compared to the respective controls (Figure 1D). We also observed a higher level of MPO in cCD compared to iCD. With regard to the infiltration pattern of CD3+ T cells into the inflamed CD tissue samples, we observed significantly higher levels in the ileum compared with the corresponding control tissue (Figure 1E). This increase was, however, not observed in the inflamed cCD samples. Interesting results were found by investigating infiltration by FoxP3+ cells in each of the three cohorts; significant differences were detected between cCD and iCD, with inflamed colon tissue showing higher FoxP3 levels than inflamed ileum tissue (Figure 1F,G). We also identified a significant increase in the percentage of FoxP3+ cells in cCD compared to colon control tissue. In ileum tissue samples, no corresponding increase of FoxP3 was detected. Expression of CD68, CD11b, CD11c, MPO, CD3 and FoxP3 were significantly increased overall in inflamed UC tissue versus controls (Figure S3). In summary, we were able to show localization-specific differences in the numbers of various infiltrating immune cells between the two CD phenotypes: In patients with iCD, percentages of both CD3+ and CD11b+ cells were elevated, whereas in patients with cCD, CD68+ and FoxP3+ cells were more abundant. The numbers of CD11c+ and MPO+ infiltration cells were elevated in both subtypes, iCD and cCD. While we successfully showed differences in the respective immunological profiles of iCD and cCD, we will focus below on enzymes and metabolites of the TRP metabolism regulated by the inflammation-associated immune cell infiltrate.

### 3.2. Inflammation-Induced IDO1 Expression Is More Pronounced in iCD Compared to cCD

**Figure 2:**
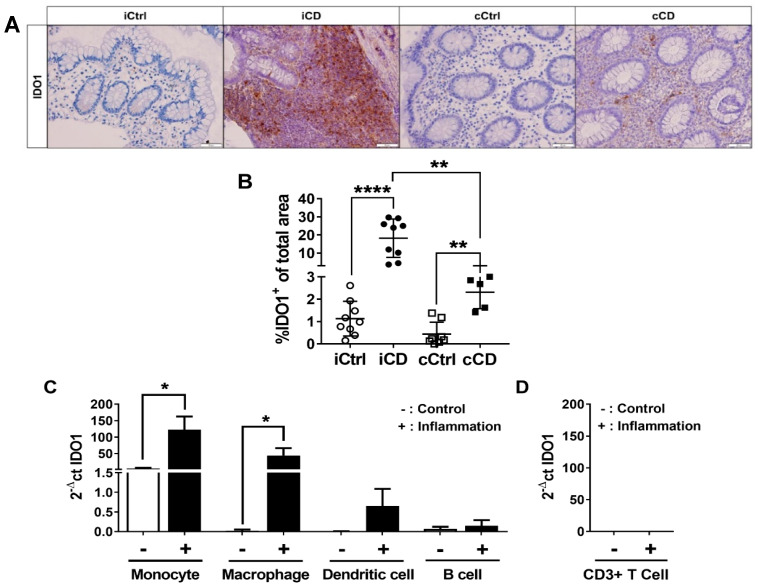
IDO1 expression is increased in the intestinal tissue of patients with active CD. (**A**) Analysis of IDO1 protein expression in paraffin-embedded tissue samples of patients with CD using IHC (representative images, magnification 20×). Inflamed tissue of patients with CD (ileum and colon) were compared to noninflamed control samples. Active inflammation was determined using the Riley score. (**B**) Differential quantitative and localization-dependent evaluation of total IDO1+ area in patients with ileal and colonic CD, compared to control samples. (**C**,**D**) Distribution and regulation of IDO1 mRNA expression in (**C**) Antigen-presenting cells and (**D**) CD3+ T cells. Antigen-presenting cells and CD3+ T cells were stimulated for 24 h with IFNγ (100 U/mL), TNFα (100 ng/mL) and IL-1β (10 ng/mL). mRNA expression levels were determined by TaqMan real-time PCR as described in Section 2. (**B**–**D**) Data are shown as mean ± SD of *n* = 3–9; * for *p* ≤ 0.05, ** for *p* < 0.01, and **** for *p* < 0.0001 using the (**B**) Mann–Whitney test or (**C**,**D**) paired Student’s *t*-test. cCD = colonic CD, cCtrl = colon non-IBD control, iCD = ileal CD, iCtrl = ileum non-IBD control.

Inflammation-induced expression of the TRP-metabolizing enzyme IDO1 has been described in several publications in the context of IBD. However, a detailed site-specific comparison of the two TRP-metabolizing enzymes IDO1 and TDO in inflamed (iCD vs. cCD) and noninflamed (iCD/cCD vs. iCtrl/cCtrl) intestinal tissue has not been performed to date. Therefore, we specifically investigated IDO1 and TDO expression in the inflamed intestinal segments of patients with CD and UC compared with noninflamed tissue from the same segments of control subjects without IBD. As expected, histological analysis showed IDO1 expression to be highly increased in the inflamed segments of patients with both CD and UC, whereas control tissues exhibited low expression. IDO1 expression was detected both in the subepithelial layer and in the crypts of the inflamed IBD samples. In contrast, in healthy non-IBD samples, IDO1 was expressed in the subepithelial layer only (Figure 2A). TDO was detectable in all the intestinal samples, independent of inflammation status (Figure S4D). In all analyzed samples, TDO was found both in the immune cell fraction and within the epithelial layer of the bowel segment. We found no significant differences in the overall TDO expression pattern between cohorts (Figure S4D–F). Comparison of iCD tissue with noninflamed ileum control samples revealed a highly significant increase of IDO1 expression in the inflamed iCD tissue (Figure 2A,B). Increased IDO1 expression was also found in the cCD samples (Figure 2A,B). However, although IDO1 expression in cCD tissue differed from that found in controls, its expression was lower than in iCD tissue (Figure 2A,B). Additionally, we analyzed the IDO1 expression pattern in patients with UC. In line with previous reports, we found a significant increase of IDO1 expression in inflamed UC versus control tissue (Figure S4A,B). Since we anticipated elevated IDO1 in the tissues to be associated with heightened immune cell infiltration, we analyzed and compared IDO1 expression in naïve and inflammatory APCs and T cells in vitro by qRT-PCR. Our results confirmed a close correlation of APC counts, especially monocytes and macrophages, with IDO1 expression (Figure 2C). A corresponding increase in IDO1 mRNA expression was determined in vitro in both cell populations after inflammatory stimulation. In contrast, CD3+ T cells showed no signal for IDO1 mRNA expression (Figure 2D). In summary, our results confirmed not only that IDO1 expression was increased in the inflamed tissue of patients with IBD, but also, remarkably, that it clearly differed in the two subtypes of CD, iCD and cCD.

### 3.3. Despite High Expression of IDO1, Local Kynurenine Levels Are Reduced in Inflamed Ileal Tissue of Patients with CD

**Figure 3:**
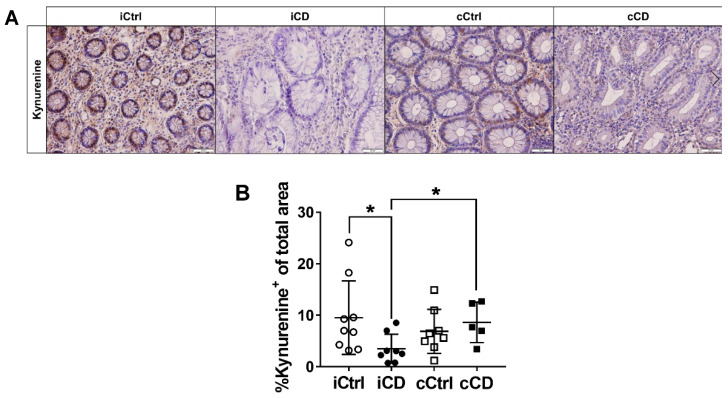
Lower kynurenine levels indicate a highly activated tryptophan metabolism in ileum tissue of patients with CD. (**A**) Analysis of local kynurenine metabolite distribution in paraffin-embedded tissue of patients with CD by IHC (representative images, magnification 20×). Inflamed tissue samples of patients with ileal and colonic CD were compared to noninflamed control samples. Active inflammation was determined by Riley score. (**B**) Differential quantitative and localization-dependent evaluation of total kynurenine+ area in patients with CD compared to controls. (**B**) Data are shown as mean ± SD of *n* = 4–9 of different patient groups and controls; * for *p* ≤ 0.05 using Mann–Whitney test. cCD = colonic CD, cCtrl = colon control, iCD = ileal CD, iCtrl = ileum control.

Previous studies focused exclusively on the enzyme expression of IDO1 and TDO. While these studies suggested Kyn to be a key factor in immune regulation, local Kyn metabolite distribution has yet to be investigated. We therefore used IHC to analyze the distribution of Kyn in small and large bowel tissue of patients with IBD. Unexpectedly, in iCD samples, we observed a local decrease of Kyn, despite concurrently high expression levels of IDO1 (Figure 3A,B). However, this effect was not found in cCD biopsies (Figure 3A,B). Comparing inflamed tissue samples of iCD versus cCD, we observed significantly lower Kyn levels in the ileum (Figure 3A,B). On the other hand, we found no clear difference in Kyn levels in inflamed UC bowel segments compared with control tissue (Figure S5A,B). Consequently, we hypothesized that enzymes downstream of IDO1 and TDO might be differentially activated in the inflamed tissue of patients with IBD, especially in iCD versus cCD.

### 3.4. Expression of Kynureninase Differs between iCD and cCD

**Figure 4:**
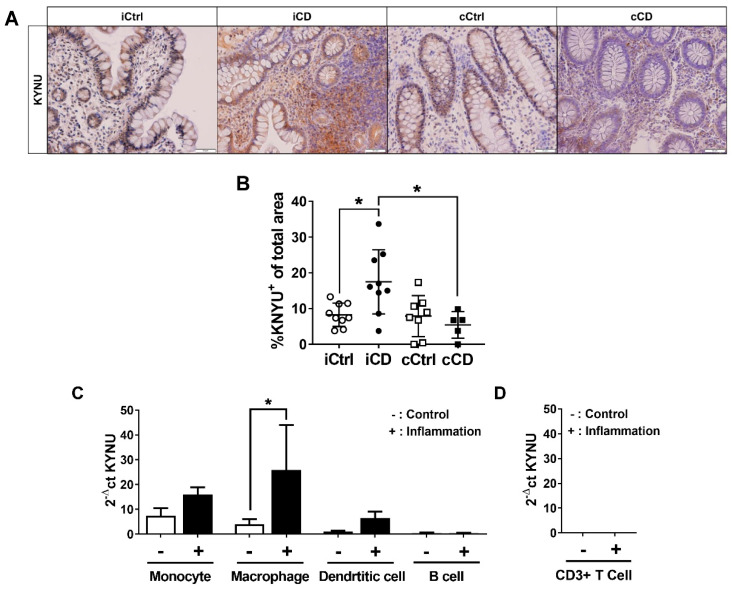
Differential kynureninase (KYNU) expression in tissue samples of patients with ileal and colonic CD. (**A**) Analysis of KYNU protein expression in paraffin-embedded tissue of CD patients via IHC (representative images, magnification 20×). Inflamed tissue of patients with ileal and colonic CD were compared to noninflamed control samples from the respective bowel segments. Active inflammation was determined by the Riley score. (**B**) Differential quantitative and localization-dependent evaluation of total KYNU+ area in CD, compared to non-IBD control (**C**,**D**) Distribution and regulation of KYNU mRNA expression in (**C**) antigen-presenting cells and (**D**) CD3+ T cells (no signal detected). Antigen-presenting cells and CD3+ T cells were stimulated for 24 h with IFNγ (100 U/mL), TNFα (100 ng/mL) and IL-1β (10 ng/mL). mRNA expression levels were determined by TaqMan real-time PCR as described in Section 2. (**B**–**D**) Data are shown as mean ± SD of *n* = 3–9; * for *p* ≤ 0.05 using the (**B**) Mann–Whitney test or (**C**,**D**) paired Student’s *t*-test. cCD = colonic CD, cCtrl = colon non-IBD control, iCD = ileal CD, iCtrl = ileum non-IBD control.

The tryptophan metabolite Kyn is degraded by two enzymes in particular: KMO, resulting in the product 3-hydoxykynurenine, and KYNU, producing anthranilic acid (AA) and 3-HAA. To determine the downstream metabolic pathways of local intestinal Kyn, we analyzed the protein expression pattern of KYNU+ area and KMO+ area in biopsies of patients with IBD. KYNU was expressed in both IBD and control samples, with intraepithelial immune cells and crypt cells showing expression of KYNU in all samples (Figure 4A and Figure S6A). KMO expression was also detected in control samples and inflamed intestinal tissue of patients with IBD (Figure S6C). Interestingly, however, KMO expression was specifically limited to crypts. In inflamed iCD, we observed a significantly greater KYNU+ area versus ileal control samples (Figure 4A,B). In contrast, no increase in the KYNU+ area was observed in colonic CD samples compared to colon controls (Figure 4A,B), whereas the percentage of KYNU+ area was significantly increased in iCD versus cCD tissue. In biopsies from patients with UC, no significant differences in the KYNU levels were observed (Figure S6A,B). Importantly, we found a negative correlation of the percentage of KYNU+ area with the percentage of KMO+ area in inflamed iCD tissue (Figure S6E). Whereas the KYNU+ area was increased in inflamed iCD samples, KMO+ area was diminished. As described above for IDO1 mRNA and protein expression, we subsequently tested the hypothesis that KYNU might be dominantly expressed in immune cells. APCs showed the strongest mRNA expression of KYNU (Figure 4C). While monocytes, macrophages and dendritic cells showed basal KYNU mRNA expression, KYNU mRNA increased only in macrophages after stimulation. T lymphocytes remained negative for KYNU mRNA (Figure 4D).

### 3.5. Kynurenine Downstream Metabolite 3-Hydoxyanthranilic Acid Is Increased in iCD

**Figure 5:**
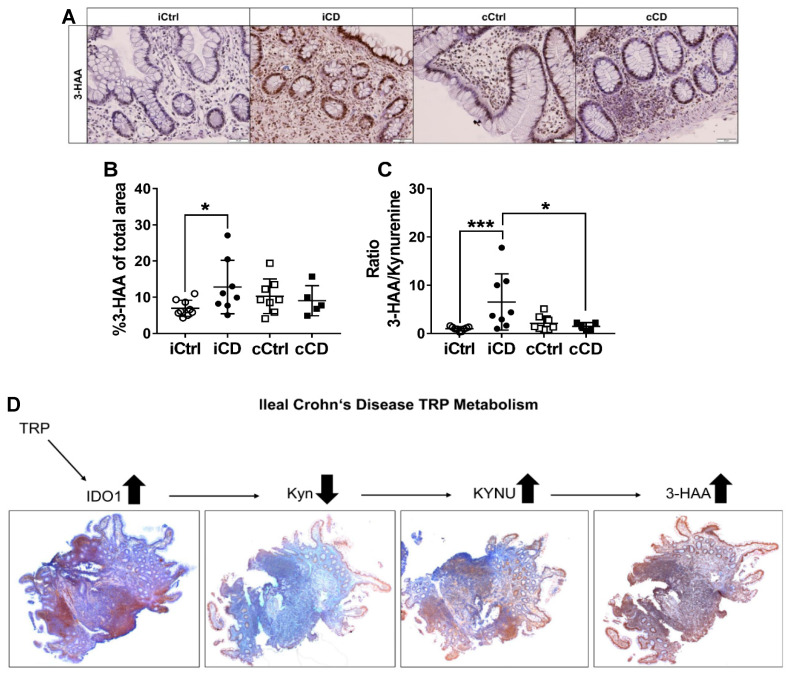
Increased 3-HAA levels in ileal CD. (**A**) Analysis of the 3-HAA metabolite distribution pattern in paraffin-embedded tissue of patients with CD via IHC (representative images, magnification 20×). Inflamed tissue of patients with ileal and colonic CD were compared with noninflamed control samples. Active inflammation was determined by the Riley score. (**B**) Differential quantitative and localization-dependent evaluation of total 3-HAA+ area in CD compared to non-IBD control. The 3-HAA/kynurenine ratio was calculated in tissue of (**C**) CD compared to non-IBD control. (**D**) Summary of the TRP enzyme and downstream metabolite expression pattern in iCD (magnification 4×). (**B**,**C**) Data are shown as mean ± SD of *n* = 4–9 for different patients and controls; * for *p* ≤ 0.05, and *** for *p* < 0.001, using the Mann–Whitney test. cCD = colonic CD, cCtrl = colon non-IBD control, iCD = ileal CD, iCtrl = ileum non-IBD control.

To date, the function of TRP downstream metabolite 3-HAA in the pathobiology of chronic inflammatory diseases is not well understood. Moreover, a specific target of 3-HAA has not yet been defined. In order to determine whether there is direct relationship between increased KYNU expression and tissue metabolite levels, most likely reflecting enzyme activity, we analyzed 3-HAA levels in inflamed intestinal tissue of patients with IBD, as well as in control (non-IBD) tissue. In both IBD and control samples, 3-HAA positive cells were found in the crypts as well as in the immune cell fraction (Figure 5A and Figure S7A). Importantly, we observed significantly higher levels of 3-HAA in iCD tissue compared to controls (Figure 5A,B). In cCD samples, we failed to detect enhanced 3-HAA levels compared to colon control tissues (Figure 5A,B). In order to indirectly evaluate the enzyme activity of KYNU, we determined the ratios of the KYNU product 3-HAA to the substrate Kyn. The ratios calculated suggest that KYNU activity was increased in iCD but not in cCD (Figure 5C). In contrast to the findings in CD samples, no differences in the expression profile of 3-HAA were identified in biopsies from patients with UC (Figure S7A,B). Figure 5D summarizes the profiles of the ileum-specific TRP enzymes and metabolites in patients with CD. In iCD, a strong increase was observed in the enzyme expression of IDO1 and KYNU, whereas Kyn levels decreased. The 3-HAA levels were also found to be enhanced. In cCD, IDO1 protein expression was increased, whereas KYNU and the metabolites Kyn and 3-HAA showed no significant differences versus the control samples.

## 4. Discussion

The aim of this work was to gain a deeper insight into the relationship between mucosal immune TRP metabolism and chronic inflammatory mechanisms in patients with IBD. In particular, we focused on characterizing new biomarkers and revealing molecular mechanisms that may represent novel potential therapeutic targets in IBD. To our knowledge, we are the first group to have identified a distinct mucosal enzyme and metabolite pattern of the local TRP metabolism in patients with IBD. Interestingly, we observed differences in the immunological profile, higher protein expression levels of the Kyn-degrading enzyme KYNU and higher levels of the 3-HAA/Kyn ratio in inflamed ileum versus inflamed colon tissue of individuals with CD. These results can be used to further classify and determine the heterogeneity of CD subtypes.

Why is it important to introduce a further subclassification of CD pathologies? Recent publications support clinical observations of different phenotypes of patients with CD depending on the primary localization of inflammation within the intestine. For example, Ahmad et al. showed specific mutations of the *CARD15* and *NOD2* genes, which only and specifically occurred in the ileum of patients with CD [7]. Naftali et al. demonstrated a correlation of enhanced presence of Fusobacterium with the disease activity of iCD by microbiotic profiling [6]. Differences in therapeutic response have also been noted between patients with CD depending on whether inflammation was predominantly localized in the ileum or in the colon. Specifically, it was proven that infliximab, a monoclonal antibody against TNFα, was associated with a better prognosis when applied in patients with cCD versus iCD [2].

Consistent with the identification of active chronic inflammation by means of the Riley score, we found an increased infiltration of immune cells in both CD and UC samples (Figure 1 and Figure S3). Our data showed that iCD pathophysiology appears to be triggered by T-lymphocyte-driven immunity, whereas cCD tissue followed a more autoinflammatory, innate immune pattern. We determined an increase of CD3+ T cells and a decrease of FoxP3+ regulatory T cells in iCD. In contrast, cCD immunological profile analysis was characterized by a significant increase of CD68+ macrophages and MPO+ neutrophil granulocyte infiltration.

Can we then link TRP metabolism to the immunological profile of patients with CD? Data from our in vitro experiments (Figure 2 and Figure 4) further substantiate the findings of other research groups [27], in suggesting that processes of the TRP metabolism can be attributed to specific cells of the immune system. APCs and granulocytes (especially neutrophils and eosinophils) are equipped with TRP-metabolizing enzymes, while T cells and T-cell-specific AHR in general represent targets of these molecules. This is corroborated by the inability of CD3+ T cells to express IDO1, TDO or KYNU, as a result of which these cells cannot produce or degrade Kyn by currently described pathways [28,29].We deliberately chose a single-cell analysis of blood cells, since this allows the clear isolation of subpopulations using FACS and the specific stimulation with cytokines, which are also involved in the course of Crohn’s disease. These in vitro data of our mRNA-based screening provided information about KYNU induction and thus the direct influence of inflammatory cytokines. Additionally, a clear assignment of enzymes to cell type was possible. In the overall concept, we were able to show an increased expression of KYNU on the protein level (IHC) as well as on the mRNA level (qPCR), which, we believe, further clarifies the role of KYNU and a possible modulation of this enzyme.

We utilized IHC screening to show that IDO1 expression is significantly increased in inflamed ileal tissue compared with inflamed colon tissue of patients with CD (Figure 2B). IDO1 is the rate-limiting enzyme of the TRP metabolism and enables the relatively inert TRP to enter the Kyn metabolism with its immunomodulatory effects. In addition, IDO1 has been described as an inflammation-inducible enzyme [30,31]. Thus, IDO1 can be understood as a general activity marker for inflammatory processes, as confirmed by the positive correlation between the Riley score and local IDO1 expression in UC samples (Figure S4). Concurrently, our results revealed very low expression levels of IDO1 in healthy bowel tissue (Figure 2B). Our data suggest that the TRP metabolism plays a far greater role in ileal than in colonic inflammation in patients with CD. Immune histological analysis also demonstrated that IDO1 and the downstream enzymes of the TRP metabolism were carried into sites of active inflammation by infiltrating immune cells. In support of this hypothesis, the majority of crypts were devoid of IDO1 protein expression. On the other hand, as we detected no significant regulation of TDO in the different intestinal segments (Figure S4), it appears to play a minor role. This is in line with Badawy et al. [22], who found that TDO exerted a systemic function by regulating TRP content in plasma, whereas IDO1, which is regulated by inflammatory cytokines produced by immune cells, such as IFNγ, TNFα or IL-1β, in turn modulated local TRP levels [22].

We investigated mucosal tissue levels of Kyn using in situ immune histochemical stainings with specific antibodies. Surprisingly, and contrary to our expectations, these measurements failed to show elevated tissue levels of Kyn (Figure 3B). In both UC and cCD samples, Kyn levels did not differ compared to controls, whereas Kyn levels were clearly reduced in iCD. Kyn has been reported to influence AHR activity in T and epithelial cells and ensure the maintenance of epithelial integrity through production of IL-22 and induction of responsiveness to IL-10 receptor [32]. Kyn also directs the differentiation of IL-10-producing Treg lymphocytes [20]. Since our analysis showed Kyn tissue levels to be decreased in patients with iCD, our data implicate that increased IDO1 expression is not automatically associated with elevated Kyn tissue levels. An IDO1/Kyn-dependent immunosuppressive milieu can thus no longer be assumed. In line with this, Treg infiltration was not significantly increased in iCD tissue compared to controls (Figure 1F).

We additionally analyzed the protein expression by analysis of KMO+ area and KYNU+ area as indirect markers of the further degradation of Kyn. KMO expression levels were generally low, whereas KYNU protein expression was moderate to high in all samples (Figure 4A). Thus, we concluded that the physiological route of Kyn degradation in the gastrointestinal system might involve accumulation of AA and 3-HAA through the metabolizing enzyme KYNU. Analogously, Giorgini et al. demonstrated in murine brain tissue that the preferred route of Kyn downstream metabolism led to the accumulation of AA and thus to enzymatic catalysis by KYNU [33]. Herein, for the first time, we determined a significantly higher expression of the Kyn-metabolizing enzyme KYNU in inflamed ileal samples from patients with CD (Figure 4B). The specific role of KYNU in inflammation pathology has not yet been clarified in sufficient detail. However, one recent study demonstrated that KYNU is strongly expressed in psoriasis [21]. Harden et al. hypothesized that KYNU regulates the transition between cancer and chronic inflammatory disease by modulating the concentrations of Kyn and other downstream TRP metabolites. Our results are in line with this hypothesis. Data clearly identified different metabolic signatures in iCD and cCD, with a simultaneous prominent presence of IDO1 and decreased levels of Kyn in the ileum, presumably resulting from the strong expression of KYNU in this segment (Figure 4B). These data are corroborated by the accumulation of 3-HAA in ileal CD biopsies (Figure 5B). Such an accumulation was observed neither in UC nor in colonic CD tissue samples (Figure 5B and Figure S7). The (patho-)physiological effect of 3-HAA is not yet well understood. While various authors have reported immunosuppressive effects of 3-HAA, others showed proinflammatory effects. However, the former were demonstrated by applying nonphysiologically high concentrations (up to 100 µM) of 3-HAA without subsequently assessing cytotoxicity [34,35,36]. In the blood of healthy volunteers, 3-HAA concentrations range from 21 to 383 nM [37]. In tissue, concentrations are expected to be even lower.

Our investigation and the derived hypothesis may be supported by measuring the ratio between 3-HAA and Kyn in future studies focusing on the differential characterization of ileal and colonic inflammation in CD. In our research, we used the 3-HAA/Kyn ratio as an indirect measurement of KYNU enzyme activity. Since this ratio addresses both the regulatory/immunosuppressive potential of Kyn and the potentially proinflammatory activity of 3-HAA, we expect it to have a greater predictive power as a CD-specific inflammatory marker than individual measurements of Kyn and 3-HAA. We found the 3-HAA/Kyn ratio to be specifically higher in inflamed iCD versus inflamed cCD tissue (Figure 5C). While a differential diagnosis of iCD and cCD by endoscopic examination is warranted, our results set the stage to establish a systemic biomarker to identify iCD. Further studies to confirm the iCD tissue-specific Kyn/3-HAA ratio by LC-MS/MS in human blood and stool samples are underway. IHC techniques allow an enzyme to be assigned to the immune cell infiltrate parallel to quantitative analysis and other pathological investigations within a single bioptic sample. Common LC-MS/MS- or qPCR-based analyses, on the other hand, do not permit the investigation of single cells and thus fail to take account of the influence of different cell types. In addition, since these methods usually expend an entire biopsy, further analyses necessitate an additional sample. For these reasons, we propose IHC to be the most effective method of investigating mucosal TRP immune metabolism.

## 5. Conclusions

In conclusion, this study demonstrates that both the overexpression of KYNU and an elevated ratio of the TRP metabolites 3-HAA/Kyn are specific to inflamed ileal tissue in CD. Our results suggest that KYNU has potential significance as a new biomarker for iCD that deserves attention in future clinical studies. In subsequent research, it would be interesting to correlate the local mucosal TRP metabolite pool with the systemic TRP metabolite pool located in the blood. Our data could represent a foundation for the development of new therapeutic approaches for CD, for example, through inhibition of KYNU with additional administration of niacin to prevent accumulation of toxic TRP metabolites, thus maintaining the energy metabolism of immune cells and mediating accumulation of the immunosuppressive metabolite Kyn. In the future, one of the key challenges in IBD will be to understand its heterogeneities and pathologies more precisely in order to develop targeted, personalized therapies. Furthermore, the identification of new, more accurate and specific biomarkers to obtain maximal clarity of diagnosis is a prerequisite for the efficient application of such tailored therapies and the minimization of unsuitable and/or superfluous medication.

## Figures and Tables

**Table 1:** Clinical features of the patient cohort and origin of biopsies. Patients with active inflammatory bowel disease (IBD) (Crohn’s disease (CD) and ulcerative colitis (UC)) were included, and histological samples from different intestinal localizations were compared with corresponding (non-IBD) control tissue samples. Histological grading was performed using the Riley score.

	Control (Ctrl)	Crohn’s Disease (CD)	Ulcerative Colitis (UC)
			
*n*	12	12	11
Age (year, average ± SD)	31 ± 11	41 ± 17	45 ± 15
Gender (f/m)	8/4	6/6	7/4
Year of diagnosis			
2015	-	11	9
2016	-	1	2
Localization (number of samples)			
small intestine	9	9	-
large intestine	12	5	11
Histological Score			
Riley score [22] (mean, range)			
total	0	3 (2–4)	2.3 (2–4)
small intestine	0	3.2 (2–4)	-
large intestine	0	2.8 (2–4)	2.3 (2–4)

[22] Activity assessed according to Riley, S.A., Gut, 1991.

## Supplementary Materials

The following are available online at https://www.mdpi.com/2077-0383/9/5/1360/s1, Table S1: Primary antibodies applied for IHC, Figure S1: Representative IDO1 staining as example for data analysis of IHC, Figure S2: Representative examples of IHC negative control stainings, Figure S3: Immune cell infiltrates in intestinal tissue of patients with active IBD, Figure S4: Quantification of TRP metabolizing enzymes IDO1 and TDO in active patients with IBD, Figure S5: Quantification of TRP metabolite kynurenine in patients with UC, Figure S6: Quantification of KYNU and KMO in active patients with IBD, Figure S7: Quantification of TRP metabolite 3-HAA in UC patients.
