# Supplementary material for: Inflammation-Induced Mucosal KYNU Expression Identifies Human Ileal Crohn’s Disease
Source: J Clin Med. 2020 May 6;9(5):1360. doi: 10.3390/jcm9051360 (PMC7290775; doi:10.3390/jcm9051360)
Supplement: Supplementary file 1 [file jcm-09-01360-s001.pdf]

Supplementary materials:

Table 1. Primary antibodies applied for IHC.

	Description	Host	Company	*Dilution*
1	monoclonal anti-CD3 (F7.2.38)	mouse	ThermoFisher, Rockford, USA	1:100
2	monoclonal anti-CD68 (KP1)	mouse	Abcam, Cambridge, UK	1:200
3	polyclonal anti-CD11b (PA5-29633)	rabbit	ThermoFisher, Rockford, USA	1:200
4	monoclonal anti-CD11c (CL1831)	mouse	Atlas Antibodies AB, Bromma, Sweden	1:150
5	monoclonal anti-FoxP3 (236A/E7)	mouse	Abcam, Cambridge, UK	1:200
6	polyclonal anti-MPO (A0398)	rabbit	Dako, Santa Clara, USA	1:300
7	monoclonal anti-IDO1 (EPR20374)	rabbit	Abcam, Cambridge, UK	1:1000
8	monoclonal anti-TDO2 (OTI2A4)	mouse	ThermoFisher, Rockford, USA	1:200
9	polyclonal anti-KYNU (HPA031686)	rabbit	Atlas Antibodies AB, Bromma, Sweden	1:200
10	polyclonal anti-KMO (8564)	rabbit	Novus Biologicals, Centennial, USA	1:2000
11	monoclonal anti-L-Kynurenine (3D4-F2)	mouse	ImmuSmol, Bordeaux, France	1:100
12	monoclonal anti-3-HydroxyAnthanilic acid (5B2-G2)	mouse	ImmuSmol, Bordeaux, France	1:500

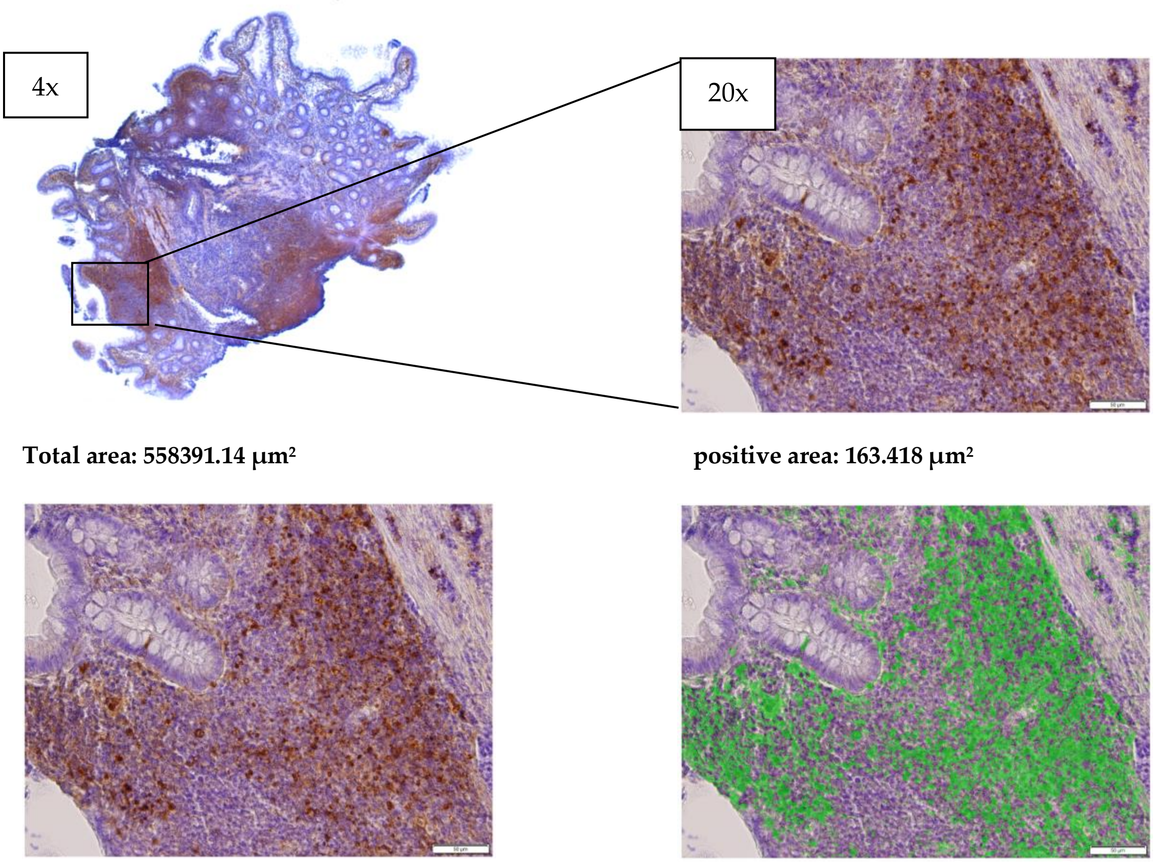

$$\frac{163.418\ (positive\ area)}{558391.14(\ total\ area)} \times 100 = 29.27\%$$

Figure S1. Representative IDO1 staining as example for data analysis of IHC. For quantitative evaluation, a suitable region was selected within the 4x magnification. This region was characterized according to active inflammation and the associated immune cell infiltration in accordance with the Riley Score[22]. Subsequently, sequential analysis was performed in the 20x magnification for any target structure. Quantification was performed with the BZ Analyzer 9000 Software from Keyence. Having been programmed to recognize which coloration was positive, the software calculated the total positive area as shown above. Both stromal cells and epithelial cells were included in the calculation. Areas in which no cells (hematoxylin positive) were present were excluded from the calculation of total area.

Secondary antibody anti-mouse

Secondary antibody anti-rabbit

small intestine

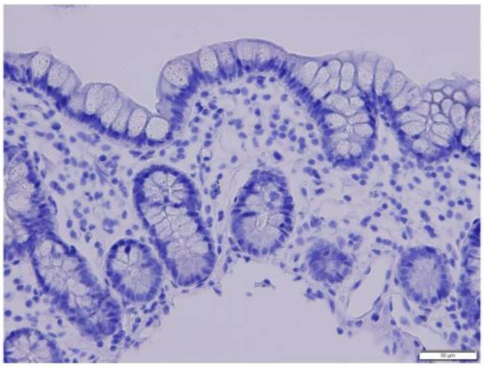

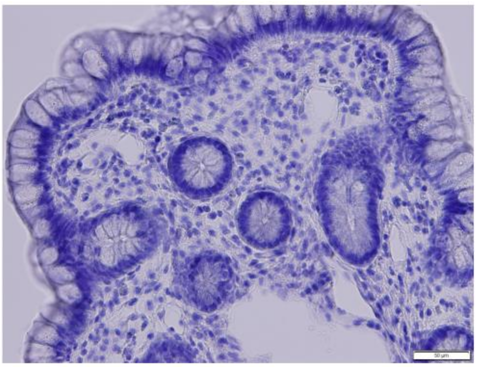

large intestine

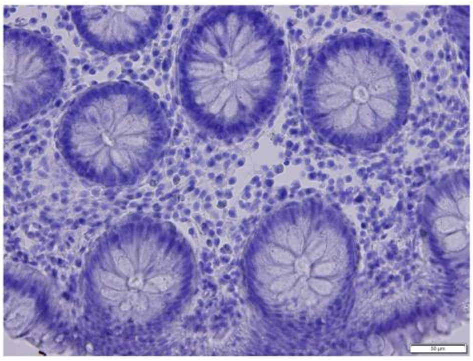

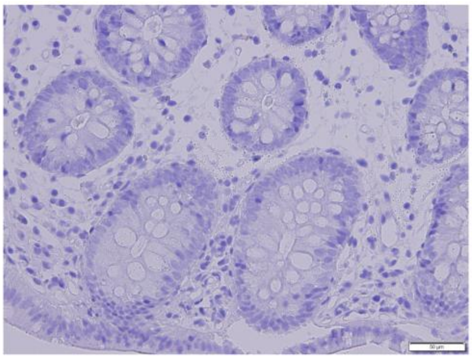

Figure S2. Representative examples of IHC negative control stainings. The complementary secondary antibody (mouse or rabbit) was carried out with each dye series without use of the respective primary antibody. As shown above, no positive signals for the corresponding target molecules were detected in the slides. All tissue locations (small intestine, large intestine) were checked according to this method. The IHC protocol is shown in Material and Methods.

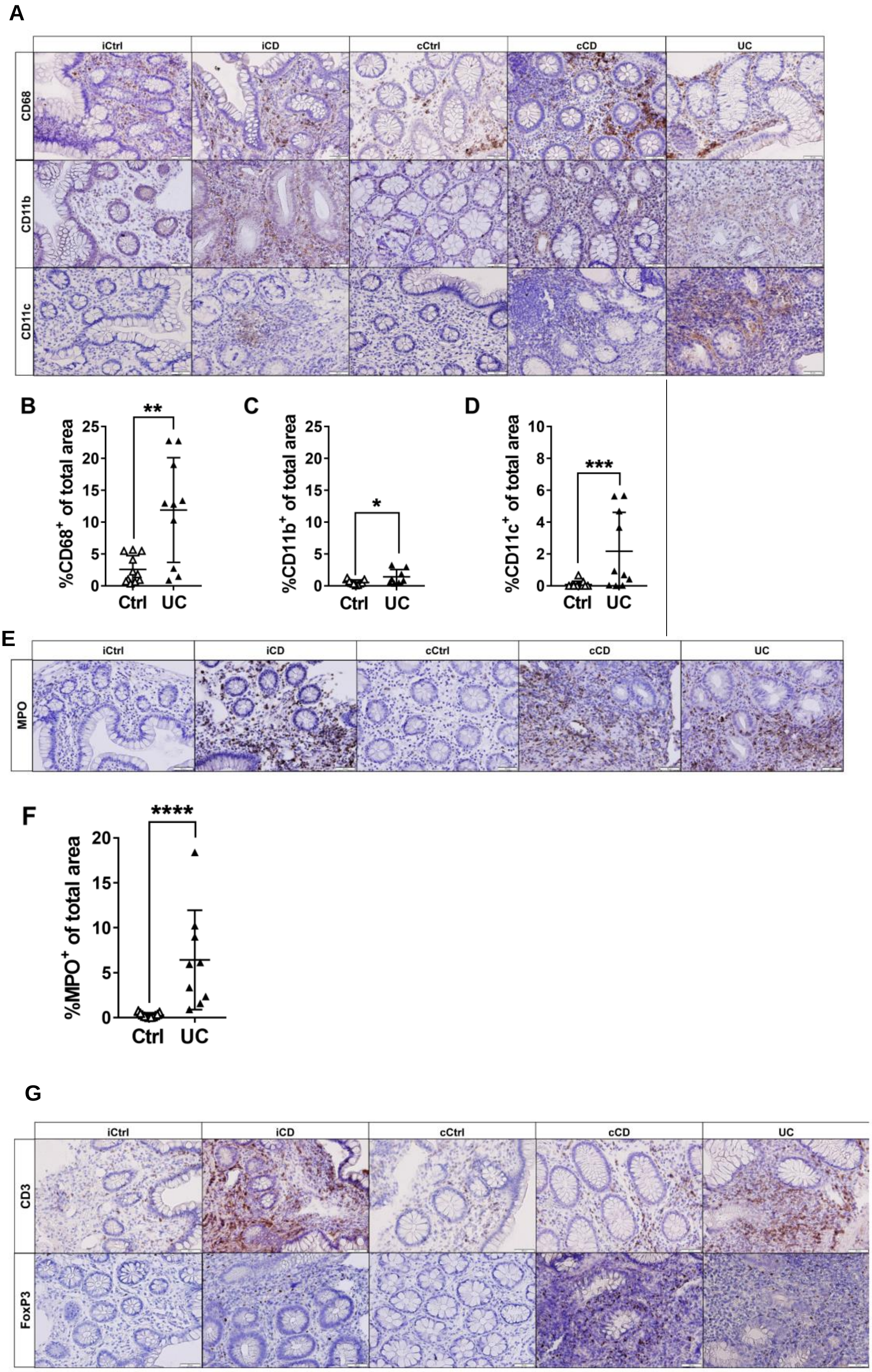
A
iCtrl
iCD
cCtrl
cCD
UC
CD68
CD11b
CD11c
B
**
%CD68+ of total area
0
5
10
15
20
25
Ctrl
UC
C
*
%CD11b+ of total area
0
5
10
15
20
25
Ctrl
UC
D
***
%CD11c+ of total area
0
2
4
6
8
10
Ctrl
UC
E
iCtrl
iCD
cCtrl
cCD
UC
MPO
F
****
%MPO+ of total area
0
5
10
15
20
Ctrl
UC
G
iCtrl
iCD
cCtrl
cCD
UC
CD3
FoxP3

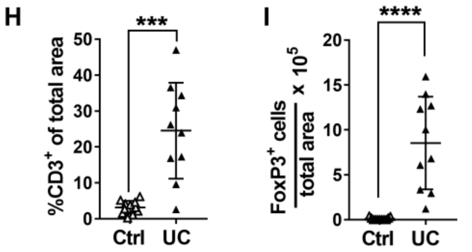

Figure S3. Immune cell infiltrates in intestinal tissue of patients with active IBD. (A, E, G) Analysis of the immune cell markers CD68, CD11b, CD11c, MPO, CD3 and FoxP3+ in paraffin-embedded tissue samples of patients with IBD using IHC (representative images, magnification 20x). Inflamed tissue of patients with ileal CD, colonic CD and UC were compared with non-inflamed control (ctrl) samples. Active inflammation was confirmed by the Riley score. Differential quantitative and localization-dependent evaluation of **(B)** total CD68+ area, **(C)** total CD11b+ area, **(D)** total CD11c+ area, **(F)** total MPO+ area, **(H)** total CD3+ area and **(I)** total FoxP3+ area. **(I)** For the analysis of FoxP3, we determined the ratio of counted cells to total area (μm^2) and multiplied with 10^5 for better visualization. **(B-D, F, H-I)** Data are shown as mean ± SD of n = 4-12 for different patient groups and controls. * for $p \leq 0.05$, ** for $p < 0.01$, *** for $p < 0.001$ and **** for $p < 0.0001$ using the Mann-Whitney test. cCD= colonic CD, cCtrl = colon non-IBD control, iCD = ileal CD, iCtrl = ileum non-IBD control, UC = ulcerative colitis

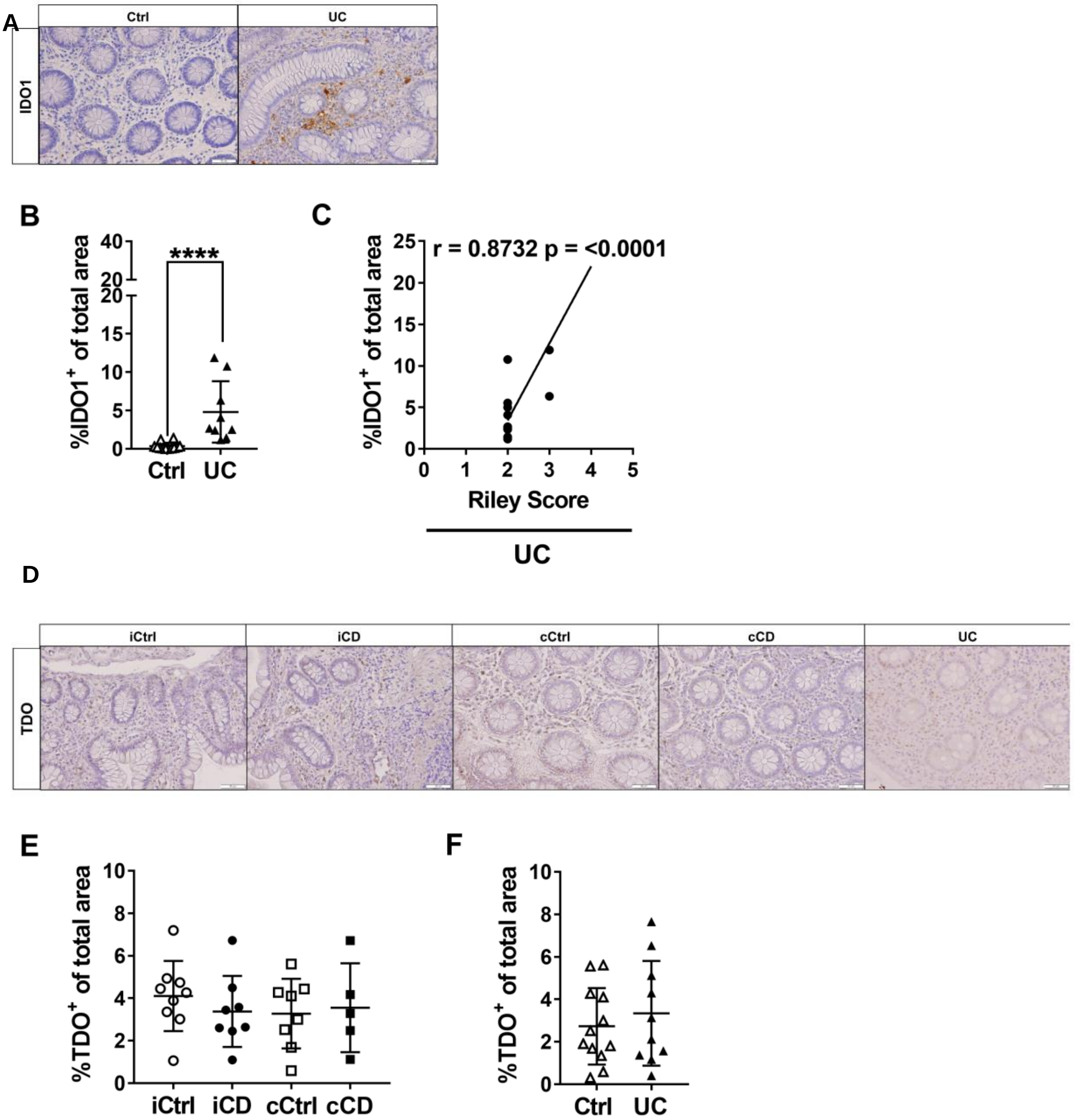

Figure S4. Quantification of TRP metabolizing enzymes IDO1 and TDO in active patients with IBD. (A, D) Analysis of IDO1 and TDO in paraffin-embedded tissue samples of patients with IBD using IHC (representative images, magnification 20x). Inflamed tissue of patients with ileal CD, colonic CD and UC were compared with non-inflamed control (ctrl) samples. Active inflammation was confirmed by the Riley score. Differential quantitative and localization-dependent evaluation of **(B)** total IDO1+ area in UC samples, **(E)** total TDO+ area in CD samples, and **(F)** total TDO+ area in UC samples were compared to the respective control samples. **(C)** Pearson correlation between Riley score and total IDO1+ area in inflamed tissue of patients with UC. **(B, D-F)** Data are shown as mean ± SD of n = 4-12 for different patient groups and controls using the Mann-Whitney test or Kruskal-Wallis test with Dunn's posttest (for C). cCD= colonic CD, cCtrl = colon non-IBD control, iCD = ileal CD, iCtrl = ileum non-IBD control, UC = ulcerative colitis

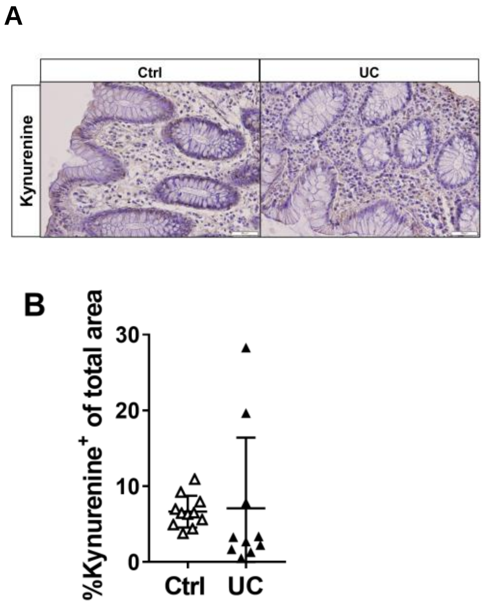

Figure S5. Quantification of TRP metabolite kynurenine in patients with UC. (A) Analysis of kynurenine in paraffin-embedded tissue samples of patients with UC using IHC (representative images, magnification 20x). Inflamed tissue of patients with UC were compared with non-inflamed control (ctrl) samples. Active inflammation was confirmed by the Riley score. Differential quantitative and localization-dependent evaluation of **(B)** total kynurenine+ area in UC samples. **(B)** Data are shown as mean ± SD of n = 10-12 for different patient groups and controls using the Mann-Whitney test. Ctrl = non-IBD control, UC = ulcerative colitis

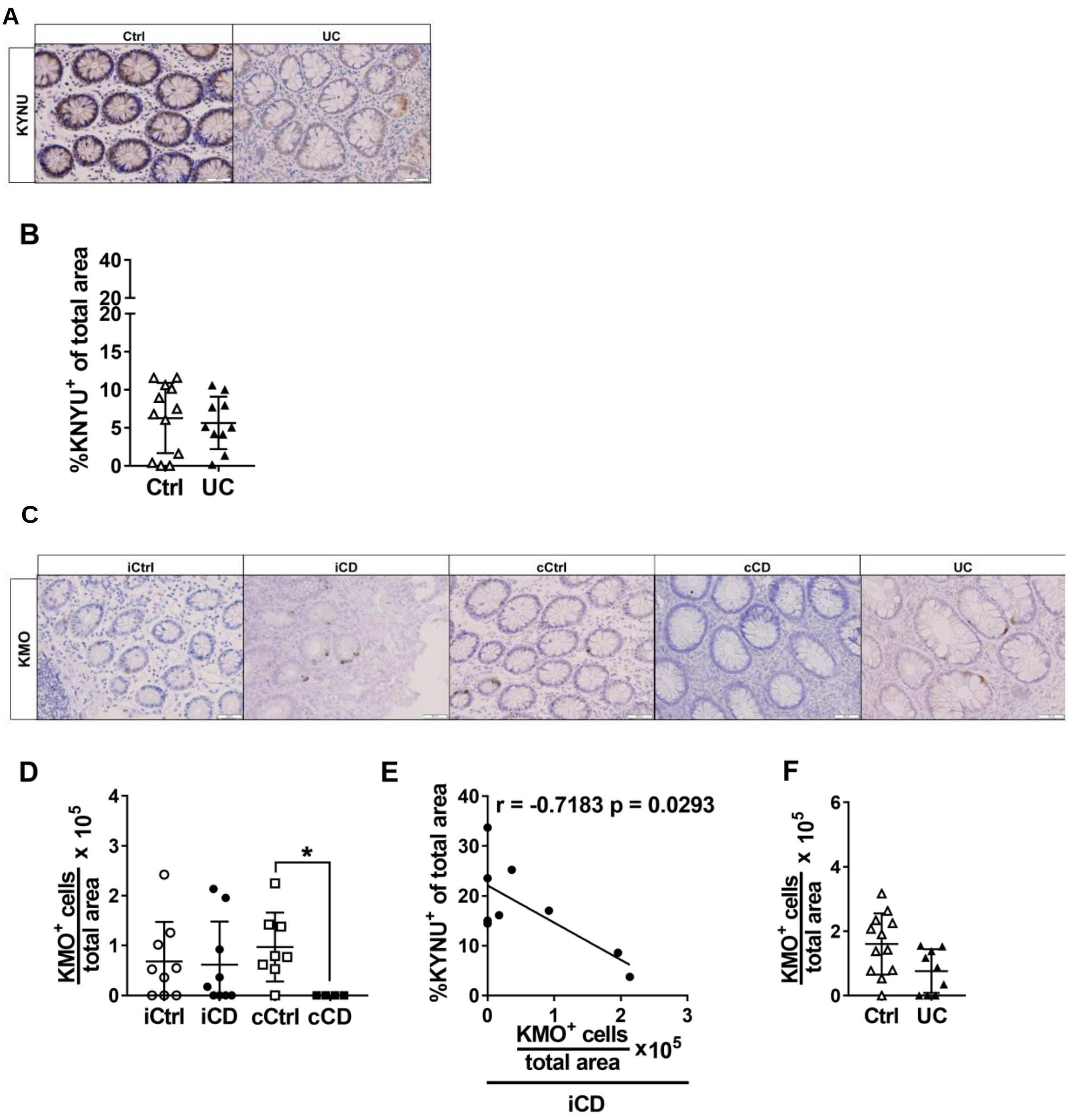

Figure S6. Quantification of KYNU and KMO in active patients with IBD. (A, C) Analysis of KYNU and KMO in paraffin-embedded tissue samples of patients with IBD using IHC (representative images, magnification 20x). Inflamed tissue of patients with ileal CD, colonic CD and UC were compared with non-inflamed control (ctrl) samples. Active inflammation was confirmed by the Riley score. Differential quantitative and localization-dependent evaluation of **(B)** total KYNU+ area in UC samples, **(D)** total KMO+ area in CD samples and **(F)** total KMO+ area in UC samples. For the analysis of KMO, we determined the ratio of counted cells to total area (μm^2) and multiplied with 10^5 for comparable illustration. In Figure S6, **(E)** Pearson's correlation coefficient was applied between total KYNU+ area and total KMO+ area in inflamed ileal tissue of CD patients. **(B, D, F)** Data are shown as mean ± SD of n = 4-12 for different patient groups and controls. * for $p \leq 0.05$ using the Mann-Whitney test. Ctrl = non-IBD control, UC = ulcerative colitis

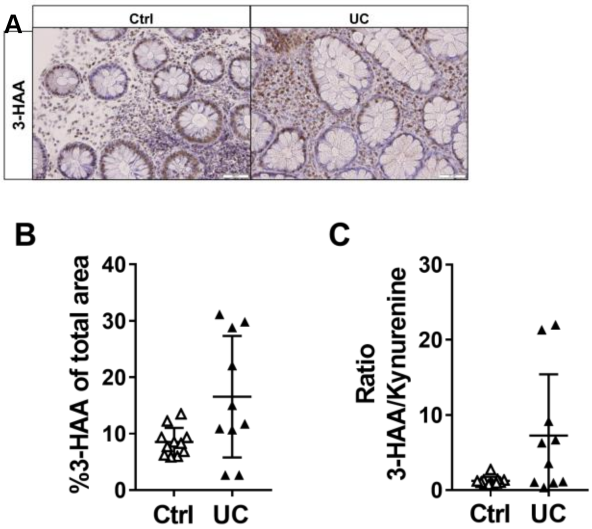

Figure S7. Quantification of TRP metabolite 3-HAA in UC patients. (A) Analysis of 3-HAA in paraffin-embedded tissue samples of patients with UC using IHC (representative images, magnification 20x). Inflamed tissue of patients with UC were compared with non-inflamed control (ctrl) samples. Active inflammation was confirmed by the Riley score. Differential quantitative and localization-dependent evaluation of **(B)** total 3-HAA+ area**.** The 3-HAA/kynurenine ratio was calculated in tissue of **(C)** UC compared to non-IBD control. **(B, C)** Data are shown as mean ± SD of n = 10-12 for different patient groups and controls using the Mann-Whitney test. Ctrl = non-IBD control, UC = ulcerative colitis
